# Value of biopsy in a cohort of children with high-titer celiac serologies: observation of dynamic policy differences between Europe and North America

**DOI:** 10.1186/s12913-020-05815-0

**Published:** 2020-10-20

**Authors:** Kamran Badizadegan, David M. Vanlandingham, Wesley Hampton, Kimberly M. Thompson

**Affiliations:** 1 Kid Risk, Inc., Orlando, FL USA; 2 Department of Pathology and Laboratory Medicine, Nationwide Children’s Hospital, Columbus, OH USA

**Keywords:** Celiac disease, Health policy, System dynamics, Value of information, Testing

## Abstract

**Background:**

Healthcare systems implement change at different rates because of differences in incentives, organizational processes, key influencers, and management styles. A comparable set of forces may play out at the national and international levels as demonstrated in significant differences in the diagnostic management of pediatric Celiac Disease (CD) between European and North American practitioners.

**Methods:**

We use retrospective clinical cohorts of 27,868 serum tissue transglutaminase (tTG) immunoglobulin A levels and 7907 upper gastrointestinal endoscopy pathology reports to create a dataset of 793 pathology reports with matching tTG results between July 1 of 2014 and July 1 of 2018. We use this dataset to characterize histopathological findings in the duodenum, stomach and esophagus of patients as a function of serum tTG levels. In addition, we use the dataset to estimate the local and national cost of endoscopies performed in patients with serum tTG levels greater than 10 times the upper limit of normal.

**Results:**

Using evidence from a US tertiary care center, we show that in the cohort of pediatric patients with high pre-test probability of CD as determined by serum tTG levels, biopsy provides no additional diagnostic value for CD, and that it counter-intuitively introduces diagnostic uncertainty in a number of patients. We estimate that using the European diagnostic algorithms could avoid between 4891 and 7738 pediatric endoscopies per year in the US for evaluation of CD.

**Conclusions:**

This study considers the North American and European management guidelines for the diagnosis of pediatric CD and highlights the slow adoption in North America of evidence-based algorithms developed and applied in Europe for triage of endoscopy and biopsy. We suggest that system dynamics influences that help maintain the *status quo* in North America include a variety of social and economic factors in addition to medical evidence. This work contributes to the growing body of evidence that the dynamics that largely favor maintaining *status quo* management policies in a variety of systems extend to clinical medicine and potentially influence clinical decisions at the level of individual patients and the population.

**Supplementary information:**

**Supplementary information** accompanies this paper at 10.1186/s12913-020-05815-0.

## Contributions to literature

This study adds to a growing body of evidence suggesting that in children with a high pre-test probability of Celiac Disease, invasive endoscopy with biopsy adds little to no additional diagnostic information with respect to Celiac Disease, and its system-wide costs may exceed any benefits.

This study provides a system dynamics framework for understanding the roots of policy differences between European and North American practitioners with respect to clinical practice guidelines.

This study adds to a growing body of literature dedicated to systems and methods for timely development and maintenance of evidence-based clinical practice guidelines and testing algorithms across the healthcare landscape.

## Background

Healthcare organizations adopt performance improvements at different rates because of differences in incentives, organizational processes, and management styles [1]. An important complicating factor in complex healthcare systems like the United States (US) is the lack of full transparency in costs, performance metrics, and clinical outcomes. As such, better or different clinical strategies may not be implemented widely, rapidly, or at all [2].

The adoption of novel practices or performance improvements may face a comparable set of resistance forces at the national or international levels. These forces accelerate the adoption of processes deemed favorable to individual providers or healthcare systems (e.g., safer medications or higher reimbursements), and decelerate the adoption of disruptive processes deemed unfavorable (e.g., elimination of revenue-generating procedures or adoption of standardized protocols). One notable example of delayed clinical implementation at the international level is the diagnostic management of pediatric Celiac Disease (CD).

The policies and positions of the European (ESPGHAN) and North American (NASPGHAN) Societies for Pediatric Gastroenterology, Hepatology and Nutrition shape the practice of pediatric gastroenterology. These sister societies frequently issue consensus guidelines, and until 2012 had equivalent diagnostic guidelines for the management of CD. In 2012, however, ESPGHAN issued a set of revised guidelines that allowed a “no-biopsy” diagnostic pathway for patients with a serum immunoglobulin A anti-tissue transglutaminase antibody (tTG) titer greater than 10 times the upper limit of normal (>10x ULN) [3]. Support for this revised position included a detailed analysis of the clinical evidence [4], the opinion of practicing physicians [5], and the results from preliminary clinical testing in a variety of conditions [6, 7].

At the time of the publication of ESPGHAN criteria, North American experts appropriately suggested that “there is still a long way to go but we are headed in the right direction” towards no-biopsy diagnosis of CD in any patient [8]. Despite multiple opportunities to reach consensus since 2012, NASPGHAN and the American College of Gastroenterology continue to maintain biopsy as a required part of the diagnosis for every suspected case of CD [9, 10]. The American Gastroenterological Association clinical update recently discussed both European and North American approach [11], but did not adopt a specific position regarding the no-biopsy approach in any patient group. Since 2012, the European experts reaffirmed and extended their position that pediatric CD can be diagnosed without biopsy in a selected group of children by following the recommended guidelines [12].

Given the substantial costs and health implications of endoscopy with biopsy in children, we explore the value of the information provided by biopsy in children with high titer serum tTG results in a large North American referral center. We show that with high pre-test probability of CD based on serum tTG values, duodenal biopsy provides no additional diagnostic value for CD, consistent with ESPGHAN findings. Moreover, biopsy counter-intuitively introduces diagnostic uncertainty in a number of patients necessitating further clinical action or follow-up. We briefly explore the economic consequences of biopsies and present a system dynamics framework to understand feedback mechanisms that enforce the *status quo* in North America. The remainder of this background provides relevant information about the pathophysiology and diagnosis of CD, including the evolution of diagnostic recommendations from by ESPGHAN and the North American response.

### Pathophysiology of CD

CD has a prevalence of 0.4–1% [13] and is in the differential diagnosis of children with any gastrointestinal symptom, particularly with predisposing conditions, including autoimmune disease, diabetes, Down syndrome, and family history [14]. Serological screening is the first line of action for evaluation of any patient with clinical suspicion of CD [3, 9, 10, 13, 15–18]. Patients with positive serology are typically referred for upper gastrointestinal (UGI) endoscopy and biopsies. Since CD is a small intestinal disease, duodenal histological abnormalities are considered the hallmark of active disease [7, 16, 17, 19–25]. Small intestinal abnormalities in CD were described in 1960s [26–31], and widespread availability of endoscopy made duodenal biopsy the *de facto* diagnostic standard. For decades, histology served as the only reliable biomarker for the disease, became known as the “gold standard,” and has remained such in spite of significant advances in laboratory testing and endoscopic imaging.

In spite of its central role in diagnosis, biopsy has well-known limitations [24, 32]. Overlap exists between histopathological findings in CD and other conditions ranging from infections to systemic disorders [24]. Writing on behalf of Gastrointestinal Pathology Society and the Association for Study of Celiac Disease, Robert et al. (2018) concluded that “correlation of histologic findings in duodenal biopsies with patient demographics, symptoms, medication use, evidence of *H. pylori* infection, and laboratory data, especially serological and genetic tests for Celiac Disease is required for correct diagnosis.” Thus, consideration of histopathology as the gold standard is not supported in practice by the need for extensive clinical correlation to reach a correct diagnosis. The widely-used Marsh histological classification acknowledges the presence of a histological spectrum, emphasizing less than perfect sensitivity and specificity of biopsy [24, 33, 34]. Importantly, all classical descriptions of CD histopathology relied on gluten-sensitivity as the definitive evidence of CD, rather than proposing the presence of pathognomonic histological features [30, 33, 35]. Pathologically, CD may show: (i) no specific histopathological findings, (ii) classical histopathology of active CD, or (iii) concurrent or superimposed confounding pathologies. Although duodenal biopsy can provide confirmation of CD *if and when* classical features are present, the overall performance characteristics of biopsy remain poorly quantified and variable because of histological overlap between multiple different inflammatory entities (reviewed in [24]).

A key issue limiting the reliability of biopsy is histological variability in tissue expression of CD [36–40]. This biological variability that can result in diagnostic uncertainty is further confounded by well-known tissue processing and interpretive errors in pathology, and biopsies in 4–30% of patients may be inadequate due to technical issues or interpretive disagreements [41–47]. Thus, recognizing that negative or non-diagnostic duodenal biopsies do not exclude CD [9, 15], practice guidelines suggest that follow-up endoscopy with additional biopsies may be justified or necessary in some patients with clinical and serological evidence of CD (i.e., high pre-test probability) for whom the laboratory reports a negative initial biopsy result [9, 14, 15, 17]. Longitudinal studies have also demonstrated histological evolution over time in patients who carry the diagnosis of CD based on clinical, serological and genetic data [48]. In these patients, duodenal histology at presentation can be non-diagnostic, suggesting that biopsy is an inherently suboptimal test in early CD.

### European movement towards no-biopsy

Acknowledging that abnormal histology is a biomarker for CD, one can appreciate the potential existence of other biomarkers (e.g., imaging, serologies or genotypes) with performance characteristics similar to, or possibly better than biopsy. Unlike histopathology, some biomarkers (e.g, genotypes) are independent of age and exposure to gluten, and therefore more generally applicable as a diagnostic tool.

An equally important concept is the probabilistic nature of all diagnostic information [49]. For example, diabetes confers 5–10% probability of CD [50], and a first-degree relative with CD is associated with 7.5% probability of CD [51]. Together, these prior probabilities imply that a patient with diabetes and an affected first-degree relative has a 7.5–16% probability of CD, depending on the level of linkage between these risk factors. Similar arguments can be made for Down syndrome, associated with CD in up to 18.6% [52], and for multiple other conditions highly correlated with CD [14, 32]. In these circumstances when the pre-test probability of CD is high, if serum tTG level rises from normal on gluten-free diet to >10x ULN after exposure to gluten, there is virtually no alternative diagnosis other than CD, regardless of any biopsy findings. The immediate utility of this probabilistic approach has been shown by others [53]. Therefore, the key policy issue is defining the population(s) in which additional testing (e.g., biopsy) provide diagnostic value *and* for which the benefits from the information exceed the costs of obtaining it [54].

Based on the Bayesian concept of essentially 100% positive predictive value for CD in a (i) symptomatic child, with (ii) serum tTG >10x ULN, and (iii) positive results of a second Celiac-specific test, ESPGHAN concluded that CD may be diagnosed without biopsy provided that (iv) signs and symptoms subside on gluten-free diet (i.e., establishment of gluten-sensitivity) [3]. These guidelines reaffirmed clinical experience suggesting that biopsy is not always necessary in patients with high pre-test probability of CD [20, 55]. The guidelines further recognize that histological variability can lead, and has led, to the need to perform multiple biopsies (with the additional procedure costs and risks) in individual patients with high-probability of CD who have indefinite or otherwise non-diagnostic biopsies at presentation [36–40].

Since 2012, the ESPGHAN no-biopsy approach has been evaluated in a variety of settings, demonstrating the overall effectiveness of the strategy [6, 22, 25, 41, 56–59]. These studies have shown opportunities for improvement, but none presented a significant challenge to the core concept that a sub-population of patients exists in which CD can correctly and confidently be diagnosed without biopsy. In one such study, the no-biopsy algorithm showed a positive predictive value of 0.988 and a negative predictive value of 0.958 [41]. This and similar recent observations [60, 61] led to reaffirmation and further extension of no-biopsy approach to include *asymptomatic* children as well [12].

### North American response

In spite of years of accumulated evidence, debate continues in the US about the adoption of *any* no-biopsy approach [10, 11, 14, 16–18, 23]. Published practice guidelines require a positive concordance between serologies and biopsy for the diagnosis of CD, and recommend obtaining multiple biopsies from distal duodenum and the duodenal bulb regardless of the pre-test probability of the disease [9, 10, 15]. A recent clinical practice guideline discussed a “biopsy-avoiding” approach and acknowledged the existence of patients in which the pre-biopsy probability of CD is “virtually 100%,” but did not specifically endorse a no-biopsy protocol [11]. Confirming the validity of the ESPGHAN guidelines in other populations has been identified as a critical need because of potential clinical differences between different patient populations [8].

An important concern raised by the proponents of an all-biopsy approach (i.e., biopsy every suspected CD case) is the uncertainty about tTG assay performance [8, 9, 14, 23, 62]. These include differences in platforms, technologies, and lack of harmonization among different laboratories that prevent cross-institutional comparison of laboratory results. Others point out a missed opportunity to diagnose incidental disorders as a disadvantage of the no-biopsy approach [8, 9, 23] without providing any formal policy, cost-benefit, or value-of-information analysis as support. Some clinicians express concern that a gluten-free diet may be cumbersome, expensive, and adversely impact the quality of life of the individual. They require confirmation of the diagnosis at the highest level of certainty before recommending a lifelong treatment [9, 62]. Thus, they implicitly value the benefits of biopsy more than its costs.

## Methods

### Clinical setting

Nationwide Children’s Hospital (NCH) is a referral center for evaluation and management of CD in the US. Since July of 2014, patients with differential diagnosis of CD have undergone tTG testing using QUANTA Flash® chemiluminescence assay (INOVA Diagnostics, Inc., San Diego, CA) which has extended analytical range (see Additional file 1) and superior performance for CD [63–65]. In addition, all duodenal biopsies at NCH are evaluated by experience pathologists and subject to clinicopathological consensus review.

### Creation of study dataset

We retrieved serum tTG IgA measured between July 1, 2014 and July 1, 2018 (27,868 tTG results). We excluded 243 adult patients (> 21 years old) and one with unknown age. Seven results with non-numeric values (assay error or cancellation) were also excluded. The remaining 27,617 results included 25,327 negatives (< 20 Chem’U), 2207 positives within reportable range (20 to 4965 Chem’U) and 83 positives higher than reportable range (> 4965 Chem’U). We did not correlate tTG levels with total IgA as this study focuses on tTG levels above the upper limit of normal, and conclusions remain independent of any potential false negative tTG values due to IgA deficiency.

We additionally retrieved pathology reports for patients with duodenal biopsy between July 1, 2014 and July 1, 2018 (7907 reports). NCH uses Marsh classification [34] for any biopsy of confirmed or suspected CD. Thus, text strings “Marsh” and/or “Celiac” in the “Final Diagnosis,” “Diagnosis Comment,” and/or “Microscopic Description” fields of pathology reports are indicative of evaluation for CD. Thus, we limited the retrieved reports to include only those with the words “Celiac” or “Marsh” in any of the above 3 fields. This yielded 895 pathology reports after excluding reports of 6 patients > 21 years of age.

The final analysis dataset was created by matching every pathology report to the nearest (in absolute time) tTG result for every unique medical record number. This excluded 96 reports without a matching tTG (patients with tTG done elsewhere and/or patients with tTG result or pathology report outside of the study period). The remaining 793 pathology reports were used for further analysis. We did not track gender or access other clinical records.

### Histopathological characterization

Histopathological findings provided by institutional pathologist in each of the 793 reports were categorized by an experienced gastrointestinal pathologist (KB). The “Celiac Disease” category included patients in whom duodenal biopsies showed increased intraepithelial lymphocytes and various degrees of villous blunting, crypt hyperplasia, and lymphoplasmacytic expansion of the lamina propria (Marsh 2 to 3c). Patients in “Indefinite Duodenitis” category either had questionable increase in intraepithelial lymphocytes with no villous blunting (Marsh 0–1), or had active or chronic duodenitis with no increase in intraepithelial lymphocytes or had confounding findings such as granulomas or marked eosinophilia. Patients in the “No Duodenitis” category had no intraepithelial lymphocytosis or other findings to suggest active or a chronic duodenitis. Cases with incidental findings not specifically associated with CD and not sufficient for a diagnosis of duodenitis were grouped under No Duodenitis. These included isolated pyloric metaplasia in duodenal bulb, focal lymphangiectasia, or mildly increased lamina propria eosinophils.

In addition to duodenal biopsies, histological findings in the stomach (786 cases) and esophagus (772 cases) were categorized. For each site, biopsies were classified as normal or abnormal, with abnormal biopsies further classified either as “significant” (unexpected and clinically actionable findings) or as “incidental” (either expected clinically actionable findings or unexpected minor findings requiring no definite clinical action).

In the stomach, significant findings included new diagnoses of *H. pylori* gastritis, or other forms of active or chronic active gastritis, including active eosinophilic gastritis and gastric ulcer. Incidental findings included any form of chronic gastritis or chronic inflammation without activity, including any reactive epithelial changes or focal metaplasia. Incidental finding also included any gastric intraepithelial lymphocytosis in the setting of CD (a known feature of CD), as well as gastritis in any patient with preoperative diagnosis of gastritis (an expected finding).

In the esophagus, significant findings included any esophagitis with greater than 8 intraepithelial eosinophils per high power field, as well as esophageal ulcers with or without fungal or viral organisms, in any patient with no preoperative diagnosis of esophagitis. In the absence of clear clinical guidelines, we considered a finding of isolated intraepithelial eosinophils (1 or 2 in a high-power field) as “normal” and eosinophil counts between 3 and 8 per high-power field as “incidental” in any patient with no preoperative diagnosis of esophagitis. We also classified occasional neutrophils with no infectious etiology and the description of increased intraepithelial lymphocytes with no specific diagnosis of esophagitis as incidental. We considered any reference to “mild reactive changes” in isolation as a normal finding. We did not encounter any other diagnostic category in the esophagus or stomach of the patients in this study of potential clinical importance for this study.

### Cost estimates

Actual cost of endoscopy with biopsy fluctuates widely based on clinical facility, insurance, type of anesthesia, level of pathology services, and multiple smaller clinical charges [66]. The actual cost of endoscopy to each patient in our study is impossible to calculate without a detailed search of the billing records, which we did not attempt because of disproportionate risk of privacy breach for the level of data obtained.

Published cost estimates are available from advocacy groups including New Choice Health™ indicating national average of $3000 per UGI endoscopy, ranging from $1600 to $12,100 [67]. For the biopsies obtained from the esophagus, stomach, duodenal bulb and duodenum per endoscopy represented by 4 charge codes in our study, pathology charges would range from $281 (average global Medicare in 2018) to $2169 (average chargemaster for two top pediatric hospitals in 2018). Separately, Miller et al. estimate $12,490 for endoscopy with biopsy [68] in a pediatric hospital. We estimate the cost of endoscopy with biopsy would range from a low of New Choice Health plus Medicare pathology ($1600 + $281 = $1881), to a high of New Choice Health plus tertiary care pathology ($12,100 + $2169 = $14,269).

Estimation of the number of pediatric procedures is equally challenging with no published data. We chose to scale NCH procedures to the national level based on the number of providers at NCH and the State population. There were 1630 pediatric gastroenterologists in the US in 2017, including 97 (6%) in Ohio [69]. Meanwhile, there were 25 pediatric gastroenterologists at NCH, representing 26% of Ohio and 1.5% of the US. Therefore, every NCH procedure scales to 3.8 in Ohio and 67 in the US. Alternatively, the number of procedures can be scaled to the national level based on US population data. US Census Data show that Ohio represented 3.6% of the US in 2018 [70]. Assuming that NCH performs 26% of pediatric gastrointestinal services in Ohio, every NCH procedure scales to 106 procedures in the US. We therefore estimate range of 67–106 procedures in the US for every NCH procedure.

## Results

### Patient characteristics

Table 1 summarizes patient characteristics as a function of serum tTG. Table 1 rows represent 2 key tTG result subgroups: Negative (< 20 Chem’U) and Positive (≥20 Chem’U), including the non-consequential designation of “Weak Positive” recommended by the assay manufacturer for positive results < 30 Chem’U. Positives are further divided into 3 categories: (i) ≥20–200 (<10x ULN), (ii) > 200 (>10x ULN), and (iii) > 2000 Chem’U (>100x ULN). The cutoff of 200 represents ESPGHAN decision point [3, 12] and 2000 Chem’U is an arbitrary cutoff because the assay has a wide analytical range spanning more than two orders of magnitude above the upper limit of normal (Additional file 1). We did not study patients with negative tTG any further.

**Table 1:** Biopsied patient characteristics as a function of serum tTG

Serum tTG (Chem’U)	Serum tTG Interpretation	Age Range (yrs)	Median Age (yrs)	Mean Age (yrs ± SD)	Total (n, %)	No Duodenitis (n, %)
Any	N/A	0.4–20.9	10.9	10.7 (± 5.0)	793 (100%)	201 (25%)
< 20	Negative	0.4–20.9	12.2	11.4 (± 5.4)	258 (32%)	141 (55%)
≥20–200	Positive	1.3–18.3	10.8	11.4 (± 4.6)	243 (31%)	8 (24%)
> 200	Positive	1.3–19.3	9.5	9.6 (± 4.8)	292 (37%)	0 (0%)
> 2000	Positive	1.5–16.7	5.8	7.3 (± 4.3)	109 (14%)	0 (0%)

### Duodenal histopathology

Every endoscopy included two sets of duodenal biopsies by protocol: one set of 4 biopsies from the distal duodenum and one set of 2 biopsies from the duodenal bulb. The percentage of endoscopies with “No Duodenitis” decreased with increasing serum tTG as expected (Table 1). None of the 292 endoscopies with serum tTG >10x ULN had normal duodenal biopsies, suggesting that high-titer tTG values are virtually diagnostic for some form of duodenal abnormality.

**Fig. 1:**
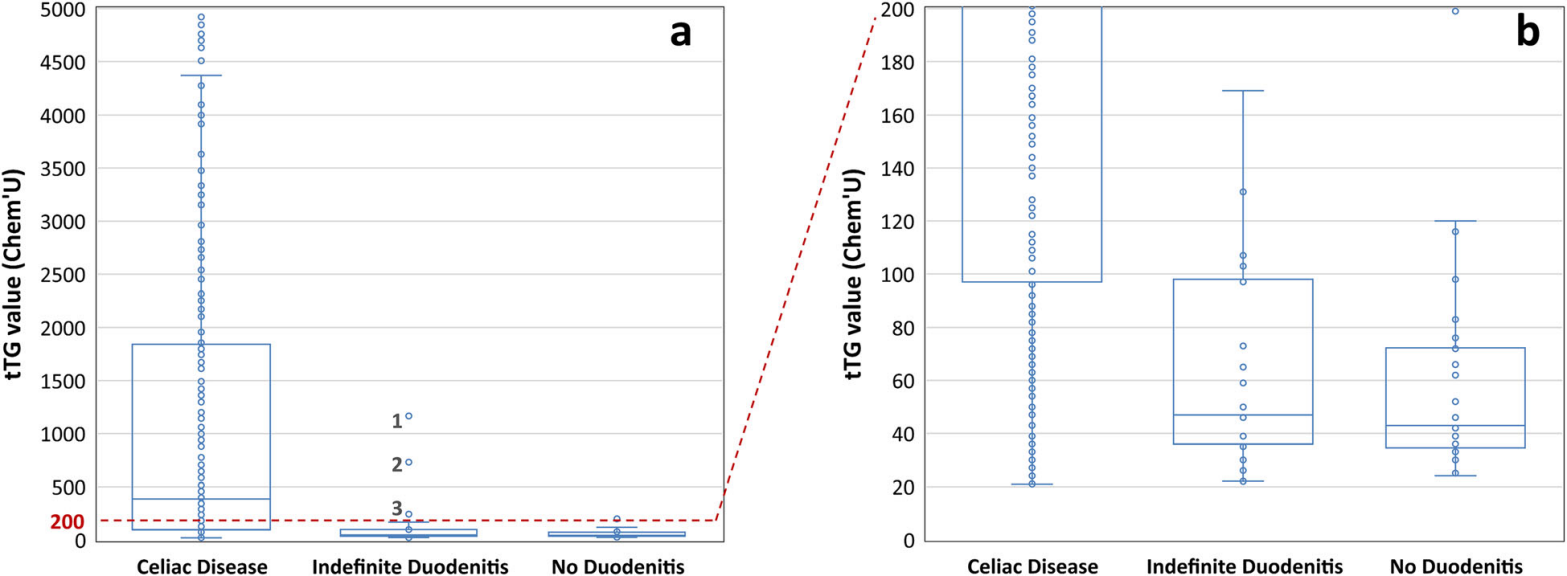
Histopathological classification of duodenal biopsies in 535 patients with positive serum tTG results. Box and whisker plot are generated using Microsoft Excel® and show the individual data (circles), as well as minimum value, first quartile, median, third quartile, maximum value, and outliers. Panel **a** shows all data, while panel **b** is limited to tTG results between 20 and 200 Chem’U. Three outliers labeled 1, 2 and 3 in the Indefinite Duodenitis group of Panel **a** are discussed in the body of the manuscript

Figure 1 expands on the distribution of tTG versus duodenal histopathology for patients with positive serology. As seen in Fig. 1(a), the upper region of the analytical range of tTG is highly associated with pathological diagnosis of CD. Applying an arbitrary cut-off of 2000 Chem’U (100x ULN), the duodenal biopsies in all 109 patients are diagnostic for CD (Marsh 2 to 3c). Applying the ESPGHAN cut-off of 200 Chem’U (10x ULN, Fig. 1a, dashed horizontal line), 289 out of 292 endoscopies (99%) are diagnostic for CD, and 3 show Indefinite Duodenitis.

We further explored the three cases with tTG >10x ULN and Indefinite Duodenitis (Fig. 1(a), data points marked 1, 2 and 3). Point #1 represents biopsies in a patient whose tTG values came down on a gluten-free diet, but did not normalize (partial sensitivity to gluten). In this patient, duodenal biopsies showed an active duodenitis, but only mild intraepithelial lymphocytosis. In addition, gastric biopsies showed focal active gastritis, raising the possibility of a superimposed process. These led to an indefinite result for CD by the pathologist. Point #2 corresponds to biopsies from a patient with tTG values completely responsive to gluten-free diet and with endoscopic abnormalities, but none of this patient’s biopsies show a specific histopathological abnormality. This patient carries a clinical diagnosis of CD, and indefinite duodenal biopsies are thought to represent a heterogenous tissue distribution, resulting in false negative histopathology. Point #3 corresponds to biopsies from a patient with Crohn’s disease proven by clinical and histopathological criteria. This patient’s tTG responds to a gluten-free diet, but biopsies are confounded by features of Crohn’s.

Figure 1(b) expands the data for endoscopies in patients with serum tTG values ranging from 20 to 200 Chem’U (positive but <10x ULN). This group shows significant uncertainty in histological correlation, and a large number of patients with positive tTG have indefinite or no histological findings diagnostic for CD. In these patients, endoscopies may provide the greatest potential diagnostic value, because a significant number of patients have no evidence of duodenitis, thus raising the possibility of false positive serology

### Esophagus and stomach

**Fig. 2:**
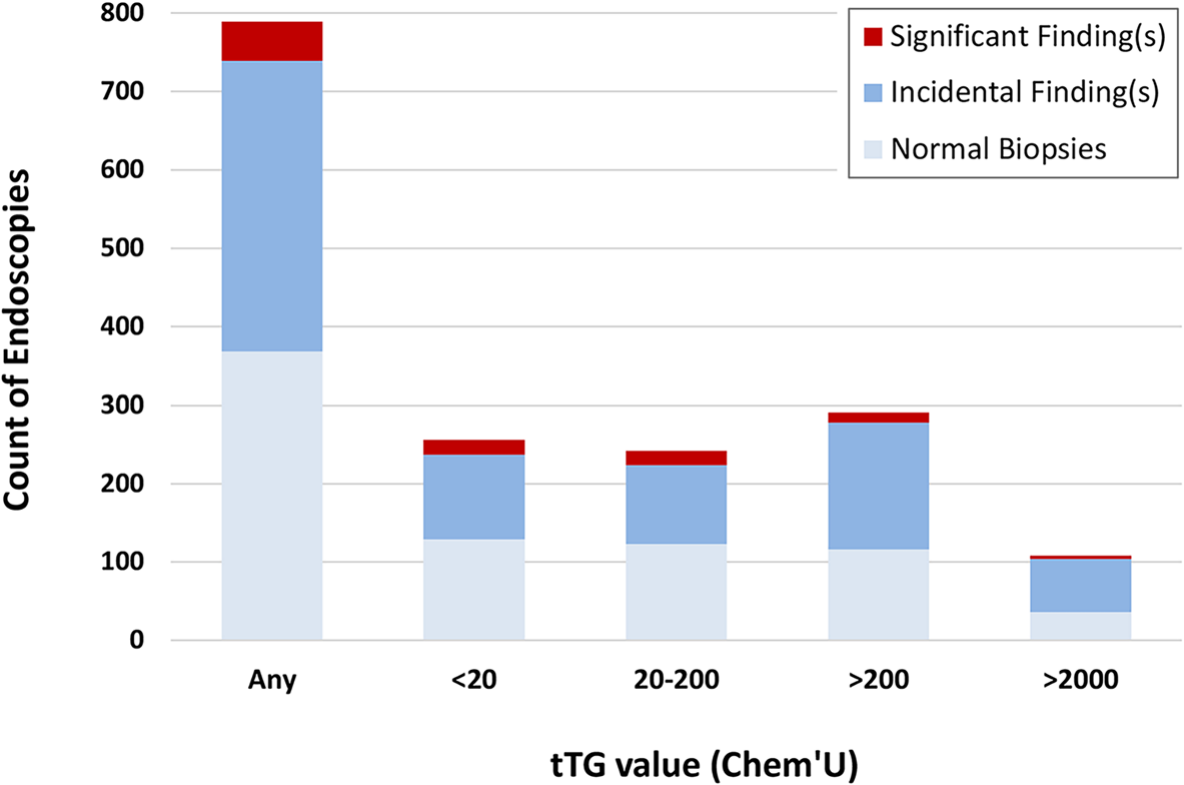
Significant and incidental findings in the esophagus and stomach as a function of serum tTG values

UGI procedures in children almost always include “protocol” biopsies of esophagus and stomach. In our dataset, 789 of 793 procedures included one or more biopsies from the esophagus (772 cases) or stomach (786 cases). Figure 2 summarizes the number of significant and incidental findings in these biopsies as a function of serum tTG.

Figure 2 shows an anticipated trend of fewer significant and incidental findings as the pre-test probability of CD goes up. In approximately 4% of procedures in patients with tTG > 200 Chem’U, there are significant (clinically actionable) findings (Fig. 2, red bars). In the 292 cases reviewed, findings included 4 *H. pylori* gastritis, 1 gastric ulcer, 1 esophageal candidiasis, 6 esophagitis with eosinophilia, and 1 esophagitis with eosinophilia and concomitant *H. pylori* gastritis. (Determination of significance is based on the limited clinical information on pathology requisitions and does not represent detailed record review.)

In contrast, we see a larger number of incidental findings (Fig. 2, blue bars), for which we cannot assess the clinical value or costs. This included 154 patients (53%) with “chronic inactive gastritis” or “chronic inflammation” (146 mild and 8 moderate), 10 patients (3%) with low-grade esophageal eosinophilia (3–8 eosinophils per high power field), and a few other incidental findings including chronic carditis, focal active gastritis and focal intestinal metaplasia. Majority of these findings, especially mild inactive gastric inflammation (146 patients), are generally non-specific and not actionable. The severity and frequency of incidental findings in our study are comparable to other diagnostic modalities [71], but our study design did not include a medical record search to determine if any of the diagnoses resulted in specific clinical action.

### Complications and costs

There were no known serious adverse events or significant pathology errors. Avoiding procedures in a population similar to the NCH population with serum tTG >10x ULN would result in local and national cost savings shown in Table 2. These estimates do include other costs to patients and their families, including pre-op and post-op clinical visits, ancillary services, lost time and wages, and delays in CD diagnosis associated with delaying adoption of a gluten-free diet.

**Table 2:** Annual health system cost savings associated with avoiding endoscopies in pediatric patients with tTG values >10x ULN (> 200 Chem’U)

	Low Cost ($1881/case)	High Cost ($14,269/case)
NCH Actual Data (73 endoscopies avoided)	$137,313	$1,041,637
US Low Estimate (4891 endoscopies avoided)	$9,199,971	$69,789,679
US High Estimate (7738 endoscopies avoided)	$14,555,178	$110,413,522

## Discussion

Development and update of clinical practice guidelines is a complex process [72–74] that may be hindered unless all stakeholders are aligned. Our studies add to a growing body of evidence that as the pre-test probability of CD increases, the value of diagnostic information progressively decreases in duodenal biopsies (Fig. 1). Our results are even more striking because unlike the ESPGHAN algorithm [3, 12], we did not include a second line of testing or consider clinical predisposing factors. Thus, the question facing North American policy makers is: What specific evidence would be required to eliminate invasive procedures in children who are effectively proven to have CD by non-invasive means?

**Fig. 3:**
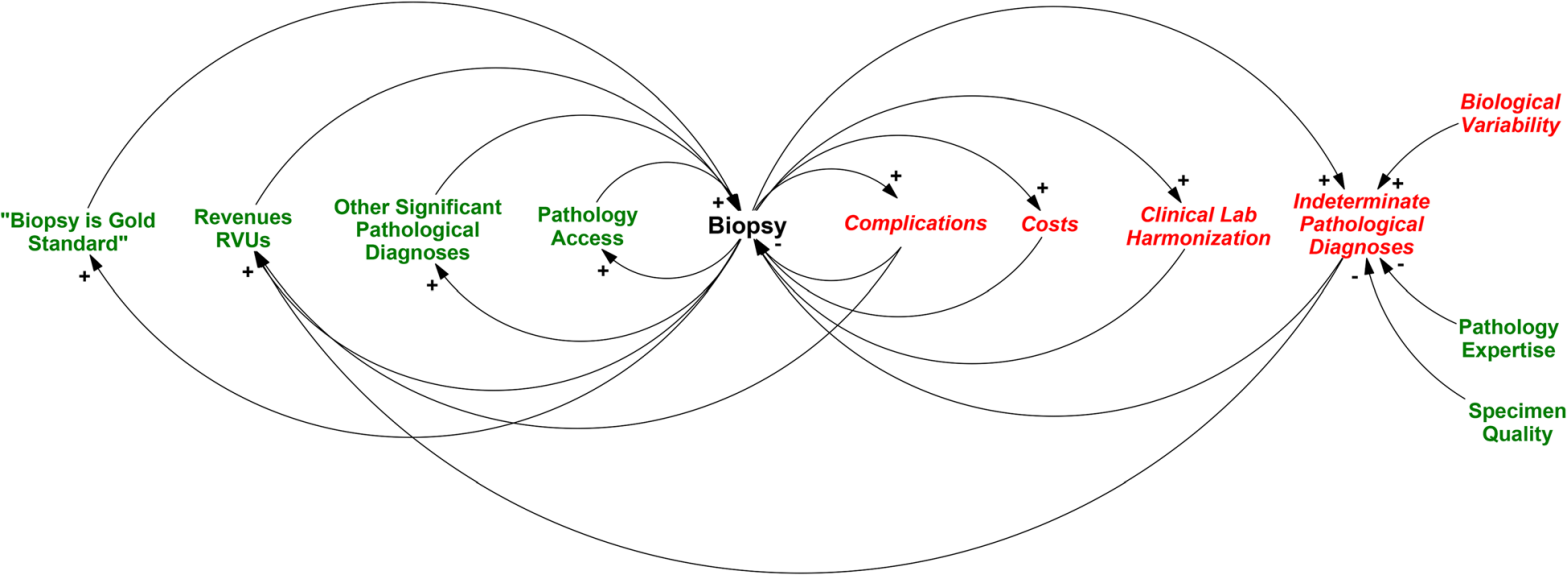
Causal loop diagram highlighting factors that could affect the decision to biopsy. Green standard font shows factors that generally reinforce the decision to biopsy. Red italic font show factors that would generally shift the balance away from doing a biopsy

With little to no value of information in biopsies for patients with high clinical probability of CD, the European guidelines present a no-biopsy pathway [3, 12], but questions remain about the barriers to adoption of *any* no-biopsy approach in America. Given the dominance of US in the North American policy decisions, the feedback loops that enforce the all-biopsy approach are likely rooted in collective medical evidence, as well as the set of beliefs, workflows, and financial incentives that actively or subconsciously shape the practice of medicine in the US. Considering a system dynamics approach [75], we observe that such system-wide forces collectively act in favor of maintaining the procedure-centric *status quo* in the US. In Fig. 3 we show a system-wide causal loop diagram highlighting potential factors that affect the decision to biopsy, which for the sake of discussion we group into three general categories: cultural, financial, and biomedical.

Cultural influences include the beliefs, assumptions, and values that underlie a given professional practice. Important practice elements that positively enforce the biopsy include the “gold standard” concept and ease of “access” to pathology with subspecialty “expertise” in the US. As discussed, the validity of biopsy as a “gold standard” remains questionable because of biological variability, lack of histopathological specificity, specimen quality issues, pathologists’ expertise, and interobserver variability. In spite of these limitations, North American practitioners maintain an absolute diagnostic role for histopathology. This position is reinforced by nearly universal access to pathology laboratories, many of which provide subspecialty service in gastrointestinal pathology, resulting in real or perceived notion of diagnostic expertise and quality. Combined with similarly accessible endoscopy services across most of North America, biopsy as a diagnostic modality is almost never a limiting factor.

Financial incentives provide substantial reinforcement for the all-biopsy approach by actively or passively shaping the clinical practice. Endoscopy and biopsies generate significant revenues for physicians and healthcare systems in North America and there is no meaningful scrutiny with respect to the delivery of value-based care. In addition, highly subspecialized healthcare services are routinely under pressure to defray investment and maintenance costs by maximizing case volume, case complexity and reimbursement rate. As such, the healthcare system costs that would normally have a negative impact on biopsy are offset by favorable revenues, while costs to patients, families and the society are ignored or downplayed.

Also reinforcing an all-biopsy approach is the belief that biopsy results in clinically valuable incidental findings that would otherwise go undiagnosed with a no-biopsy approach. These additional diagnoses become positive externalities representing a perceived win-win situation for all parties involved. In support of these arguments, we show that 4% of procedures in patients with tTG values > 200 Chem’U result in a clinically actionable histopathological finding in the esophagus or stomach (Fig. 2). While these “freebies” seem valuable to the system, they represent an optimism bias that highly values potential (albeit rare) actionable findings without consideration of their cost to patients and the healthcare system. The idea that endoscopy is an effective screening method for incidental identification of upper gastrointestinal disease is not an accepted idea in clinical practice. In symptomatic children who undergo diagnostic UGI procedures, the yield for any positive diagnosis is less than 40% [76]. Our case series, which represents one of the largest pediatric endoscopy studies to date, shows that truly unexpected clinical findings in the stomach and esophagus occur in approximately 6% for all comers and less than 4% in patients with high pre-test probability of CD (Fig. 2). Furthermore, these small percentage of clinically actionable and unsuspected diagnoses come at the expense of a large number of incidental findings that may stigmatize patients with an “abnormality” of no known significance and may lead to additional follow-up testing, including follow-up endoscopies to document resolution of findings. Lastly, the contention that clinically significant diagnoses would go undetected but for the endoscopy for CD remains unproven. Patients with eosinophilic esophagitis or *H. pylori* gastritis routinely receive diagnoses based on clinical signs and symptoms, and will receive endoscopy as necessary for those conditions. The suggestion that routine endoscopy is not necessary in some patients for the diagnosis of CD does not preclude endoscopy in these patients.

Perhaps one of the most under-emphasized factors in unconditional recommendation to biopsy is the concept of indeterminate pathological diagnoses that are either biological (i.e., patchy disease or evolving disease) or technical (i.e., poor biopsies or lack of expertise). These uncertainties are manifest in the well-known correlation between the number of biopsies and the diagnosis of CD, with doubling of the rate of diagnosis as the number of biopsies double [36]. This phenomenon also underlies the NASPHGAN requirement to obtain multiple biopsies from distal duodenum and the duodenal bulb [9, 10, 15], and the need for repeat endoscopy is acknowledged globally when the first set of biopsies are indeterminate [14, 17]. Technical quality issues limit evaluation in approximately 10% of cases [45], and the true rate and overall cost of repeat UGI endoscopy with biopsy secondary to indeterminate biopsies remains unknown. At NCH, 24% of all UGI endoscopies and 7% of endoscopies reported in Table 1 represent repeat endoscopies, but our analysis cannot determine how many of the repeats (if any) occurred for indeterminate biopsies.

Lack of laboratory harmonization represents an oft-cited reason for the need to biopsy, and it is one of the most important sources of variability and uncertainty in the diagnosis of CD. Serological assays for CD are neither standardized nor harmonized between laboratories in North America. Multiple different assays and assay technologies are used to measure tTG, and existing data do not allow or facilitate physician efforts to calibrate or “harmonize” one laboratory’s test results against another. The assumption that a positive serum in laboratory A would result in a positive result in laboratory B appears reasonable, specialty for highly abnormal results, but exceptions do occur. In addition, the important quantitative relationship between various positive results represented in Fig. 1 in our dataset remains unknown across North American laboratories. Thus, the probabilistic relationship between tTG and histopathological diagnosis shown in Fig. 1 may not directly apply to other assay technologies with different analytical sensitivity or dynamic range (see Additional file 1). ESPGHAN effectively addressed this issue by choosing a relative cut-off value expressed in “upper limit of normal.” However, clinical laboratory harmonization remains a critical need and challenge in the management of CD [77].

Finally, a critical issue that has not received as much attention is the fundamental problem of categorizing a continuous variable (serum tTG) into categories (positive and negative) (generally reviewed in [78]). When considering all “positive” serum tTG values together as a single entity, biopsy becomes necessary for a reliable classification of CD (Fig. 1). However, when viewing serum tTG as a quantitative test with a wide dynamic range (data from Table 1 depicted graphically in Additional file 1), we can easily appreciate the direct relationship between tTG and the probability of CD. In fact, the 100% probability of histologically confirmed CD in our study for tTG values greater than 2000 Chem’U (Fig. 1, panel A) demonstrates the high positive predictive value of the test in this range. Significant recent advances have increased the analytical range for serum tTG by 2–3 orders of magnitude [64], resulting in superior diagnostic performance in the evaluation of CD [63, 65]. Timely adoption and implementation of these technological advances into medical management is necessary for maintaining best-practice guidelines.

Complication rates represent another factor that can influence the decision to biopsy. However, in our experience and the experience of others [79, 80], complications related to UGI endoscopy requiring unanticipated medical attention remain very rare. In a recent study of nearly 10,000 pediatric endoscopies, Kramer and Narkewicz identified a total of 160 (1.67%) complications that resulted in additional medical evaluation and costs, none of which resulted in significant morbidity or mortality (unplanned surgery, ICU admission, or death) [81]. The long-term health impacts of UGI endoscopy in children, if any, remain unknown.

Our studies are limited by the absence of actual cost data for the cohort of patients described here. Our clinical results are also limited by the absence of detailed chart reviews required to determine the nature of follow up in each patient. These limitations do not affect the main clinical conclusions regarding the value of biopsy in patient with high tTG titers, and we believe detailed chart and billing reviews in this large cohort of children has privacy risks that are higher than any potential benefit to the study.

## Conclusions

Clinical management of children with CD provides an informative case study of medical management and policy making between Europe and North America. Starting from the same set of literature and evidence, they reach different conclusions about adoption of diagnostic evidence. We propose that factors that underlie national policy positions go beyond medical evidence, and include system-wide and often hidden or subconscious cultural and economic factors (Fig. 3) that influence decision to biopsy or not.

We acknowledge lack of laboratory harmonization as a significant obstacle in implementation of standard diagnostic algorithms. This issue was avoided in our clinical study by relying only on one assay, but given the large number screening tests for CD, global laboratory harmonization is urgently needed to enable cross-institutional studies that are necessary for development of national practice guidelines. Harmonization is technologically feasible [82, 83], but requires strong policy incentives, which professional societies can demand from laboratories (for example, consider the precedents set by Cystic Fibrosis Foundation and Children’s Oncology Group).

Another obstacle to change is favorable reimbursement structure and ease of access to specialty care resulting in an implicit bias toward performing procedures. Based on individual incentives, practitioners may over-emphasize the value of factors that favor biopsy (access to subspecialty pathology and value of incidental findings), and de-emphasize costs to the system (procedure cost and cost of managing indeterminate results) and costs to patients (time missed from work and school).

While complication rates for pediatric endoscopy are low, the risk of clinical adverse events is not zero. Furthermore, histopathology carries adverse events, including lost or mislabeled specimens and interpretive errors. Combining these costs with financial costs associated with procedures, the value of information obtained by biopsy in thousands of patients who meet the European no-biopsy criteria appears qualitatively less than the costs. Moreover, over-emphasis on the role of biopsy undermines movement towards clinical laboratory harmonization that could reduce current uncertainty in laboratory diagnosis of CD.

In summary, we suggest that system-wide factors that result in the continued practice of an all-biopsy approach in North America go beyond medical evidence and include a complex set of social and economic factors. Individual practitioners who face an ever-changing and increasingly complex environment tend to err on the side of caution which naturally translates into multiple tiers of testing before a child is diagnosed with CD. However, invasive procedures in children come with non-zero risks of adverse events, as well as multiple hidden costs to patients, families, and the healthcare system. In order to make better decisions about the diagnostic management of CD across a heterogenous collection of health care systems and laboratories, critical need exists for clinical laboratories to standardize or at least harmonize CD biomarkers such as serum tTG results. This will help eliminate a frequently cited obstacle in making informed policy decisions regarding the need for additional diagnostic testing such as biopsy. Finally, price and cost transparency are necessary requisites in continued assessment of best-practice guidelines to determine when any given diagnostic procedure cost exceeds its value of information. With increasing healthcare expenditures and complexities, we hope this study motivates discussions about systems thinking that could potentially resolve current policy differences in pediatric CD, and in general enable timely adoption and implementation of cost-effective and evidence-based clinical guidelines.

## Supplementary information

**Additional file 1.** Demonstration of differences in dynamic range (Analytical Measurement Range or AMR) for different clinical assays commonly used to measure serum tTG IgA levels.(PDF 934 kb)

## Data Availability

The datasets generated and/or analyzed during the current study are not publicly available due to the fact that they contain sensitive and/or protected health information, but are available from the corresponding author on reasonable request.

## Abbreviations

CD: Celiac Disease
Chem’U: Chemiluminescence Units (unit of measure for QUANTA Flash® assay)
ESPGHAN: European Society for Pediatric Gastroenterology, Hepatology and Nutrition
IgA: Immunoglobulin A
tTG: Tissue transglutaminase (serology)
NASPGHAN: North American Society for Pediatric Gastroenterology, Hepatology and Nutrition
NCH: Nationwide Children’s Hospital
UGI: Upper gastrointestinal endoscopy
ULN: Upper limit of normal
US: United States

## References

[1] Pisano GP, Bohmer RMJ, Edmondson AC (2001). Organizational differences in rates of learning: evidence from the adoption of minimally invasive cardiac surgery. Manag Sci.

[2] Christensen HB, Floyd E, Maffett M. The only prescription is transparency: The effect of charge-price-transparency regulation on healthcare prices. Manage Sci. 2020;66(7):2861-82. 10.1287/mnsc.2019.3330.

[3] Husby S (2012). European Society for Pediatric Gastroenterology, Hepatology, and nutrition guidelines for the diagnosis of coeliac disease. J Pediatr Gastroenterol Nutr.

[4] Giersiepen K (2012). Accuracy of diagnostic antibody tests for coeliac disease in children: summary of an evidence report. J Pediatr Gastroenterol Nutr.

[5] Ribes-Koninckx C (2012). Coeliac disease diagnosis: ESPGHAN 1990 criteria or need for a change? Results of a questionnaire. J Pediatr Gastroenterol Nutr.

[6] Kurppa K (2012). Utility of the new ESPGHAN criteria for the diagnosis of celiac disease in at-risk groups. J Pediatr Gastroenterol Nutr.

[7] Mubarak A (2012). Tissue transglutaminase levels above 100 U/mL and celiac disease: a prospective study. World J Gastroenterol.

[8] Hill ID, Horvath K (2012). Nonbiopsy diagnosis of celiac disease: are we nearly there yet?. J Pediatr Gastroenterol Nutr.

[9] Hill ID (2016). NASPGHAN clinical report on the diagnosis and treatment of gluten-related disorders. J Pediatr Gastroenterol Nutr.

[10] Rubio-Tapia A (2013). ACG clinical guidelines: diagnosis and management of celiac disease. Am J Gastroenterol.

[11] Husby S, Murray JA, Katzka DA (2019). AGA clinical practice update on diagnosis and monitoring of celiac disease - changing utility of serology and histologic measures: expert review. Gastroenterology.

[12] Husby S (2020). European society Paediatric gastroenterology, Hepatology and nutrition guidelines for diagnosing coeliac disease 2020. J Pediatr Gastroenterol Nutr.

[13] Bibbins-Domingo K (2017). Screening for celiac disease: US preventive services task force recommendation statement. JAMA.

[14] Hill, I. D. (2019, updated 3 June, 2019). Diagnosis of celiac disease in children. UpToDate [online]. Retrieved 6 Oct, 2019 from https://www.uptodate.com/contents/diagnosis-of-celiac-disease-in-children.

[15] Hill ID (2005). Guideline for the diagnosis and treatment of celiac disease in children: recommendations of the north American Society for Pediatric Gastroenterology, Hepatology and nutrition. J Pediatr Gastroenterol Nutr.

[16] Maglione MA (2016). Diagnosis of Celiac Disease, in Comparative Effectiveness Reviews.

[17] Bai JC, Ciacci C (2017). World gastroenterology organisation global guidelines: celiac disease. J Clin Gastroenterol.

[18] Chou R (2017). Screening for celiac disease: evidence report and systematic review for the US preventive services task force. JAMA.

[19] Burgin-Wolff A, Mauro B, Faruk H (2013). Intestinal biopsy is not always required to diagnose celiac disease: a retrospective analysis of combined antibody tests. BMC Gastroenterol.

[20] Donaldson MR (2008). Strongly positive tissue transglutaminase antibodies are associated with Marsh 3 histopathology in adult and pediatric celiac disease. J Clin Gastroenterol.

[21] Fernandez-Banares F (2012). Are positive serum-IgA-tissue-transglutaminase antibodies enough to diagnose coeliac disease without a small bowel biopsy? Post-test probability of coeliac disease. J Crohns Colitis.

[22] Gidrewicz D (2015). Evaluation of the ESPGHAN celiac guidelines in a north American pediatric population. Am J Gastroenterol.

[23] Reilly NR (2018). Coeliac disease: to biopsy or not?. Nat Rev Gastroenterol Hepatol.

[24] Robert ME (2018). Statement on best practices in the use of pathology as a diagnostic tool for celiac disease: a guide for clinicians and pathologists. Am J Surg Pathol.

[25] Werkstetter KJ (2017). Accuracy in diagnosis of celiac disease without biopsies in clinical practice. Gastroenterology.

[26] Rubin CE (1960). Studies of celiac disease. I. The apparent identical and specific nature of the duodenal and proximal jejunal lesion in celiac disease and idiopathic sprue. Gastroenterology.

[27] Rubin CE (1960). Studies of celiac disease. II. The apparent irreversibility of the proximal intestinal pathology in celiac disease. Gastroenterology.

[28] Padykula HA (1961). A morphologic and histochemical analysis of the human jejunal epithelium in nontropical sprue. Gastroenterology.

[29] Cameron AH (1962). Duodeno-jejunal biopsy in the investigation of children with coeliac disease. Q J Med.

[30] Yardley JH (1962). Celiac disease. A study of the jejunal epithelium before and after a gluten-free diet. N Engl J Med.

[31] Himes HW, Adlersberg D (1958). Pathologic changes in the small bowel in idiopathic sprue: biopsy and autopsy findings. Gastroenterology.

[32] Volta U, Villanacci V (2011). Celiac disease: diagnostic criteria in progress. Cell Mol Immunol.

[33] Marsh MN (1992). Gluten, major histocompatibility complex, and the small intestine. A molecular and immunobiologic approach to the spectrum of gluten sensitivity (‘celiac sprue’). Gastroenterology.

[34] Oberhuber G, Granditsch G, Vogelsang H (1999). The histopathology of coeliac disease: time for a standardized report scheme for pathologists. Eur J Gastroenterol Hepatol.

[35] Katz AJ, Falchuk ZM (1978). Definitive diagnosis of gluten-sensitive enteropathy. Use of an in vitro organ culture model. Gastroenterology.

[36] Lebwohl B (2011). Adherence to biopsy guidelines increases celiac disease diagnosis. Gastrointest Endosc.

[37] Bonamico M (2004). Patchy villous atrophy of the duodenum in childhood celiac disease. J Pediatr Gastroenterol Nutr.

[38] Prasad KK (2010). The frequency of histologic lesion variability of the duodenal mucosa in children with celiac disease. World J Pediatr.

[39] Ravelli A (2010). How patchy is patchy villous atrophy?: distribution pattern of histological lesions in the duodenum of children with celiac disease. Am J Gastroenterol.

[40] Weir DC (2010). Variability of histopathological changes in childhood celiac disease. Am J Gastroenterol.

[41] Wolf J (2017). Validation of antibody-based strategies for diagnosis of pediatric celiac disease without biopsy. Gastroenterology.

[42] Arguelles-Grande C (2012). Variability in small bowel histopathology reporting between different pathology practice settings: impact on the diagnosis of coeliac disease. J Clin Pathol.

[43] Weile B (2000). Interobserver variation in diagnosing coeliac disease. A joint study by Danish and Swedish pathologists. APMIS.

[44] Corazza GR (2007). Comparison of the interobserver reproducibility with different histologic criteria used in celiac disease. Clin Gastroenterol Hepatol.

[45] Collin P (2005). Antiendomysial and antihuman recombinant tissue transglutaminase antibodies in the diagnosis of coeliac disease: a biopsy-proven European multicentre study. Eur J Gastroenterol Hepatol.

[46] Risdon RA, Keeling JW (1974). Quantitation of the histological changes found in small intestinal biopsy specimens from children with suspected coeliac disease. Gut.

[47] Webb C (2011). Accuracy in celiac disease diagnostics by controlling the small-bowel biopsy process. J Pediatr Gastroenterol Nutr.

[48] Auricchio R (2019). Progression of celiac disease in children with antibodies against tissue transglutaminase and normal duodenal architecture. Gastroenterology.

[49] Hsu J (2011). Application of GRADE: making evidence-based recommendations about diagnostic tests in clinical practice guidelines. Implement Sci.

[50] Craig ME (2017). Prevalence of celiac disease in 52,721 youth with type 1 diabetes: international comparison across three continents. Diabetes Care.

[51] Singh P (2018). Global prevalence of celiac disease: systematic review and meta-analysis. Clin Gastroenterol Hepatol.

[52] Pavlovic M, Berenji K, Bukurov M (2017). Screening of celiac disease in Down syndrome - old and new dilemmas. World J Clin Cases.

[53] Ermarth A (2017). Identification of pediatric patients with celiac disease based on serology and a classification and regression tree analysis. Clin Gastroenterol Hepatol.

[54] Badizadegan K, Thompson KM (2011). Value of information in nonfocal colonic biopsies. J Pediatr Gastroenterol Nutr.

[55] Barker CC (2005). Can tissue transglutaminase antibody titers replace small-bowel biopsy to diagnose celiac disease in select pediatric populations?. Pediatrics.

[56] Koletzko S (2017). No need for routine endoscopy in children with celiac disease on a gluten-free diet. J Pediatr Gastroenterol Nutr.

[57] Donat E (2016). ESPGHAN 2012 guidelines for coeliac disease diagnosis: validation through a retrospective Spanish multicentric study. J Pediatr Gastroenterol Nutr.

[58] Klapp G (2013). Celiac disease: the new proposed ESPGHAN diagnostic criteria do work well in a selected population. J Pediatr Gastroenterol Nutr.

[59] Trovato CM (2015). Are ESPGHAN “biopsy-sparing” guidelines for celiac disease also suitable for asymptomatic patients?. Am J Gastroenterol.

[60] Bogaert L (2020). Optimization of serologic diagnosis of celiac disease in the pediatric setting. Autoimmun Rev.

[61] Rozenberg O (2020). Automated analyzers are suited for diagnosing celiac disease without a biopsy. J Pediatr Gastroenterol Nutr.

[62] Fasano, A., Children may not need a biopsy for celiac disease diagnosis in BeyondCeliac.org Research News. 2017: BeyondCeliac.org, Ambler, (Accessed 19 June 2019).

[63] Aita A (2013). Chemiluminescence and ELISA-based serum assays for diagnosing and monitoring celiac disease in children: a comparative study. Clin Chim Acta.

[64] Cinquanta L, Fontana DE, Bizzaro N (2017). Chemiluminescent immunoassay technology: what does it change in autoantibody detection?. Auto Immun Highlights.

[65] Lakos G (2014). Analytical and clinical comparison of two fully automated immunoassay systems for the diagnosis of celiac disease. J Immunol Res.

[66] Helmers RA (2017). Overall cost comparison of gastrointestinal endoscopic procedures with endoscopist- or anesthesia-supported sedation by activity-based costing techniques. Mayo Clin Proc Innov Qual Outcomes.

[67] Anon (2020). “Upper GI Endoscopy Cost and Procedure Information.” New Choice Health.

[68] Miller SM, Goldstein JL, Gerson LB (2011). Cost-effectiveness model of endoscopic biopsy for eosinophilic esophagitis in patients with refractory GERD. Am J Gastroenterol.

[69] ABP (2018). Pediatric Physicians Workforce Data Book.

[70] Anon. State population totals and components of change: 2010-2019. Washington, DC: U.S. Census Bureau, Population Division; 2019..

[71] Lumbreras B, Donat L, Hernandez-Aguado I (2010). Incidental findings in imaging diagnostic tests: a systematic review. Br J Radiol.

[72] Woolf S (2012). Developing clinical practice guidelines: types of evidence and outcomes; values and economics, synthesis, grading, and presentation and deriving recommendations. Implement Sci.

[73] Shekelle P (2012). Developing clinical practice guidelines: reviewing, reporting, and publishing guidelines; updating guidelines; and the emerging issues of enhancing guideline implementability and accounting for comorbid conditions in guideline development. Implement Sci.

[74] Eccles MP (2012). Developing clinical practice guidelines: target audiences, identifying topics for guidelines, guideline group composition and functioning and conflicts of interest. Implement Sci.

[75] Sterman J (2000). Business dynamics : systems thinking and modeling for a complex world.

[76] Thakkar K (2014). Outcomes of children after esophagogastroduodenoscopy for chronic abdominal pain. Clin Gastroenterol Hepatol.

[77] Stern M, for the Working Group on Serologic Screening for Celiac Disease (2000). Comparative evaluation of serologic tests for celiac disease: a European initiative toward standardization. J Pediatr Gastroenterol Nutr.

[78] Fernandes A (2019). Why quantitative variables should not be recoded as categorical. J Appl Math Physics.

[79] Tringali A (2016). Complications in pediatric endoscopy. Best Pract Res Clin Gastroenterol.

[80] Samer Ammar M (2003). Complications after outpatient upper GI endoscopy in children: 30-day follow-up. Am J Gastroenterol.

[81] Kramer RE, Narkewicz MR (2016). Adverse events following gastrointestinal endoscopy in children: classifications, characterizations, and implications. J Pediatr Gastroenterol Nutr.

[82] Plebani M, Graziani MS, Tate JR (2018). Harmonization in laboratory medicine: Blowin’ in the wind. Clin Chem Lab Med.

[83] Tozzoli R, Villalta D, Bizzaro N (2017). Challenges in the standardization of autoantibody testing: a comprehensive review. Clin Rev Allergy Immunol.

