# Supplementary material for: Value of biopsy in a cohort of children with high-titer celiac serologies: observation of dynamic policy differences between Europe and North America
Source: BMC Health Serv Res. 2020 Oct 20;20:962. doi: 10.1186/s12913-020-05815-0 (PMC7576777; doi:10.1186/s12913-020-05815-0)
Supplement: Supplementary file 1 — Additional file 1. Demonstration of differences in dynamic range (Analytical Measurement Range or AMR) for different clinical assays commonly used to measure serum tTG IgA levels.(PDF 934 kb) [file 12913_2020_5815_MOESM1_ESM.pdf]

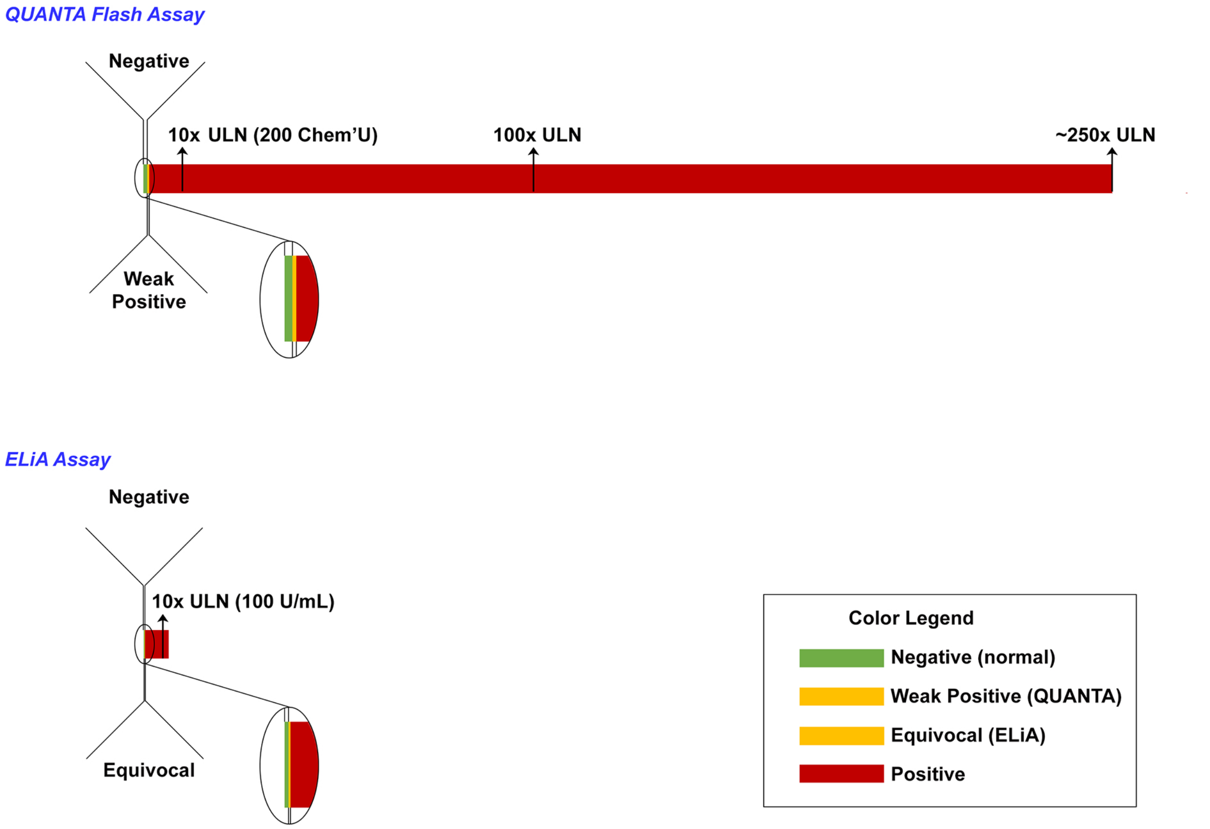

Additional File 1 - Demonstration of differences in dynamic range (Analytical Measurement Range or AMR) for different clinical assays commonly used to measure serum tTG IgA levels. *QUANTA Flash* (INOVA Diag.) is the chemiluminescence assay used in this manuscript. *ELiA* (Thermo Fisher) is typical of fluorescence assays used by multiple clinical laboratories in North America. Elliptical insets highlight the small regions of negative and weak/equivocal results that are not readily appreciated at scale. *QUANTA* and *ELiA* dynamic ranges are drawn at the same scale and represent each assay's AMR (0.1-128 U/mL for *ELiA and* 1.9-4965 Chem'U for *QUANTA*). Note that the dynamic range of chemiluminescence *QUANTA* is >2 orders of magnitude larger than fluorescence *ELiA,* enabling significant discrimination of test results, specially at levels greater than 10x the upper limit of normal (ULN). In addition, vastly different positive ranges (red bars) make it virtually impossible to "harmonize" or calibrate one assay's results to the other without losing information. (For more information about these two assays see Lakos et al. Analytical and Clinical Comparison of Two Fully Automated Immunoassay Systems for the Diagnosis of Celiac Disease. J Immunol Res; 2014:371263. doi:10.1155/2014/371263.)
